# Can the tumor neural niche be targeted to re-program cancer?

**DOI:** 10.1371/journal.pbio.3003266

**Published:** 2025-07-21

**Authors:** Erica K. Sloan, Joo Sang Lee

**Affiliations:** 1 Monash Institute of Pharmaceutical Sciences, Monash University, Parkville, Victoria, Australia; 2 Department of Precision Medicine, School of Medicine and Department of Artificial Intelligence, Sungkyunkwan University, Suwon, Republic of Korea; Princeton University, UNITED STATES OF AMERICA

## Abstract

Interactions between the peripheral nervous system and solid tumors influence cancer progression and treatment response. This Perspective argues that defining the 3D tumor neural niche using spatial omics and AI technologies could identify new opportunities for targeted therapies to stop cancer progression.

## Teaser

Interactions between the peripheral nervous system and solid tumors influence cancer progression and treatment response. Defining the 3D tumor neural niche using spatial omics and AI technologies will identify new opportunities for targeted therapies to stop cancer progression.

## Introduction

**Fig 1:**
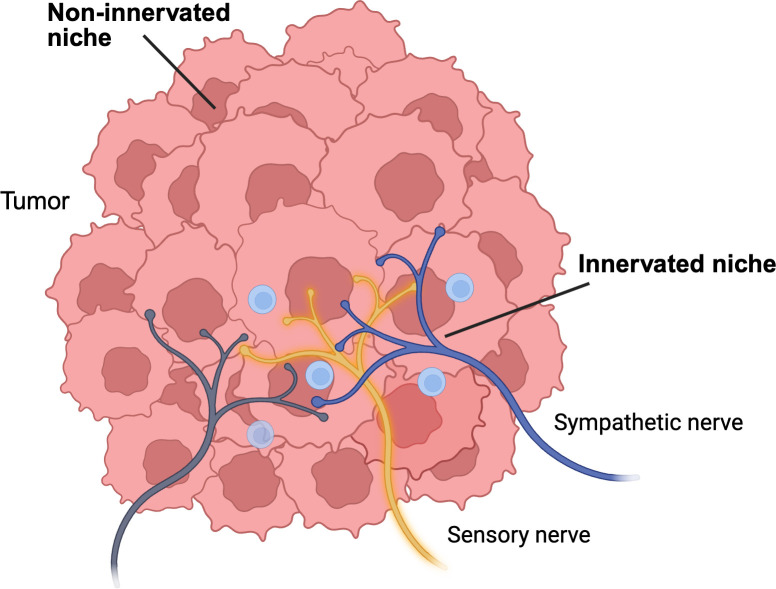
The tumor neural niche. The 3D neuronal architecture of solid tumors may support the formation of local microdomains that support immune evasion and metastatic behavior by tumor cells (pink). Immune cells: blue. Created in Biorender under Creative Commons Attribution 4.0 International (CC BY 4.0) License.

Solid tumors are innervated by the autonomic and somatosensory nervous systems. Nerves enter tumors with the vasculature and branch into the tumor parenchyma, releasing neurotransmitters and neuropeptides that modulate the behavior of tumor cells within the tumor microenvironment (TME) (Fig 1). For example, norepinephrine from the sympathetic nervous system (SNS) induces cytoskeletal changes in tumor cells and increases transcription of matrix-degrading proteases, which collectively increase the invasive behavior of tumor cells. Neuronal signaling also influences the behavior of non-malignant cells within the TME; both SNS neurotransmitters and sensory neuropeptides affect immune cell function, impairing immunosurveillance and supporting cancer progression [1,2].

Due to the wired nature of neurons innervation establishes a physical scaffold within tissues including tumors. One implication of this structural feature of innervation is that neurons may not have a uniform impact on the TME. Rather, localized release of neurochemical mediators may create distinct niches that drive immune evasion and metastatic dissemination of tumor cells. Targeting the 3D neural network within tumors could therefore offer a novel strategy to reshape the TME from the inside, disrupting the niches that sustain tumor progression.

To define the tumor neural niche, it will be important to understand the complexity of innervation within tumors. Most studies published to date have focused on defining the impact of a single neuronal population, for example, sympathetic neurons [1]. However, most tumors are likely to contain different types of neurons, reflecting the neuronal composition of the organ colonized by the tumor. In the breast, for example, the coordinated actions of sympathetic and sensory nerves control lactation. Although both of these types of neurons have been documented in breast cancers, future studies that define the coordinated actions of these different cell populations within breast cancers will be essential to holistically appreciate neural control of the breast TME.

Fully understanding the tumor neural niche will also require characterization of neuronal subpopulations and understanding of how the neural architecture of tumors changes throughout the course of disease. Recent single-cell RNA sequencing (scRNAseq) studies have identified substantial diversity among tumor-innervating sensory neuron subtypes [3]. Subtype-specific differences in neurochemical profiles will differentially impact nearby cells, highlighting the complexity of neural signaling within the TME. Furthermore, our own recent preclinical studies found that standard cancer treatments, including chemotherapy, can increase the density of innervation within solid tumors [4]. If the extent of neural regulation of the TME changes during the course of treatment, single time point snapshots will be insufficient to define the dynamic nature of tumor innervation.

Elucidating how the neural niche shapes the 3D structure and organization of the TME will also be important. Research to date has focused on cellular-level effects of neural signaling by defining how tumor cells and non-malignant cells respond to neurotransmitters and neuropeptides. Defining how these functional changes play out in the spatial context of the tissue will allow us to define how neurons coordinate cellular responses that shape the TME. We anticipate that these tissue-level effects will allow neurons to control the extent and nature of intratumor heterogeneity, which contributes to therapeutic resistance and poor prognosis [5].

To begin the process of understanding the tumor neural niche, we need to integrate neuroscience techniques with spatial cancer biology approaches. Application of these approaches to clinical samples and preclinical cancer models will be essential to define 3D neural-tumor interactions. Spatial omics technologies are now complementing high-resolution microscopy imaging approaches to map the influence of tumor-innervating neural networks on adjacent cell populations including non-malignant cells. Recent spatial transcriptomic analyses lend support to the idea that tumor innervation exerts localized effects on the surrounding environment. scRNAseq analyses of spatially-resolved laser micro-dissected pancreatic tumors have revealed transcriptional reprogramming of tumor cells as they invade nerve-rich regions during perineural invasion [6]. These observations suggest that neural proximity may induce discrete cellular states that shape distinct niches within the tumor.

To date, studies that map neuronal populations within tumors have relied on fluorescent neuronal reporter mice and immunostaining of clinical samples, coupled with microscopy analysis. While there is already a wealth of hematoxylin and eosin (H&E) stained tumor sections in clinical cancer repositories, a significant barrier to progress is the technical challenge of accurately localizing innervation within histological images or spatial omics datasets. Although larger nerve bundles are easily identified in histological sections, individual unmyelinated nerve fibers remain largely undetectable in standard H&E-stained samples but likely have a key role in shaping the neural niche as neurotransmitter release occurs from varicosities along the length of the fiber. Moreover, neuronal transcripts are sparsely distributed in innervated tissues since the bulk of neuronal RNA resides in distant ganglia rather than in axonal projections within the tumor site. Many current spatial transcriptomic platforms lack the resolution to capture the fine-grained interactions between individual nerve fibers and the adjacent cells.

Overcoming these challenges will require a multi-faceted technological approach. High-resolution spatial transcriptomic platforms that are compatible with neuron-specific immunostaining can help localize individual nerve fibers while simultaneously capturing molecular signatures in the surrounding cellular context. In parallel, new methods such as Trace-n-Seq combine retrograde tracing of neurons that project to the tumor with scRNAseq of labeled neuronal cell bodies that are located in ganglia outside the tumor [3], providing new opportunities to define the influence of tumor-innervating circuits.

Artificial intelligence (AI) also holds great potential to transform this field. AI approaches can now infer the spatial transcriptome from routinely collected H&E-stained histopathology slides, potentially unlocking new insights from vast archives of clinical samples [7]. Furthermore, advances in 3D imaging technologies such as light sheet microscopy and micro-computed tomography (μCT) are enabling reconstruction of the spatial architecture of tissues, including patterns of innervation, with unprecedented resolution [3,8]. These developments have led to AI models that predict 3D spatial transcriptomics distributions from volumetric tissue imaging [9].

Furthermore, advances in understanding the functional impact of the 3D neural architecture on the tumor are likely to arise from integrating neuroscience tools into preclinical cancer models. For example, chemo- and optogenetic modulation can be used to precisely manipulate specific neuronal populations. To map the effect of the neural niche on intratumoral heterogeneity, these approaches could be combined with cellular barcoding technologies that track the in vivo movement of individual cancer clones over time to define how neural inputs shape clonal evolution within tumors [1,5].

From a clinical perspective, defining the tumor neural niche could identify vulnerable regions for localized drug delivery and guide neuromodulatory strategies such as denervation and bioelectric modulation to reprogram the TME from within. These interventions could be warranted in cases where a tumor cannot be surgically removed. Denervation using surgical or chemical strategies is currently used for pain management and to treat renal disease. While denervation offers a way to remove neuronal inputs to a tumor, there may be benefits from electroceutical interventions that instead dial down neuronal inputs. Bioelectric devices are currently used for deep brain and vagal nerve stimulation to treat neurological and somatic diseases, and hold promise for treating cancer.

In addition to their potential as stand-alone treatments, interventions that modulate the tumor neural niche could be used to re-shape the TME to increase sensitivity to standard cancer treatments. For example, targeted modulation of the SNS could be leveraged to fine-tune anti-cancer immunity to improve response to standard therapies. SNS signaling halts immune cell motility, which limits the recruitment of immune cells to organs, impairing anti-cancer T cell responses [1]. Modulating the SNS can shift the immune profile of tumors in patients [10]; systemic pharmacological blockade of SNS signaling increased recruitment of antigen-presenting cells and CD8 T cells to breast cancers, suggesting neural modulation could be used to prime the TME to improve the efficacy of subsequent treatments.

As spatial omics and microscopy technologies improve, the development of multimodal foundation models that integrate these data types will provide a powerful way to decode the TME, including its neuronal components. Such integrative platforms will not only enhance our understanding of neural-tumor interactions but also support the rational development of novel therapeutic interventions, such as bioelectrical devices, that modulate tumor-innervating circuits. Using spatial omics and imaging technologies to map the 3D tumor neural niche will accelerate the discovery of new therapeutic targets directed at neural elements within the TME.

## Abbreviations

AI: artificial intelligence
H&E: hematoxylin and eosin
scRNAseq: single-cell RNA sequencing
SNS: sympathetic nervous system
TME: tumor microenvironment
μCT: micro computed tomography

## References

[1] Devi S, Alexandre YO, Loi JK, Gillis R, Ghazanfari N, Creed SJ, et al. Adrenergic regulation of the vasculature impairs leukocyte interstitial migration and suppresses immune responses. Immunity. 2021;54(6):1219-1230.e7. doi: 10.1016/j.immuni.2021.03.025 33915109

[2] Balood M, Ahmadi M, Eichwald T, Ahmadi A, Majdoubi A, Roversi K, et al. Nociceptor neurons affect cancer immunosurveillance. Nature. 2022;611(7935):405–12. doi: 10.1038/s41586-022-05374-w 36323780 PMC9646485

[3] Thiel V, Renders S, Panten J, Dross N, Bauer K, Azorin D, et al. Characterization of single neurons reprogrammed by pancreatic cancer. Nature. 2025;640(8060):1042–51. doi: 10.1038/s41586-025-08735-3 39961335 PMC12018453

[4] Chang A, Botteri E, Gillis RD, Löfling L, Le CP, Ziegler AI, et al. Beta-blockade enhances anthracycline control of metastasis in triple-negative breast cancer. Sci Transl Med. 2023;15(693):eadf1147. doi: 10.1126/scitranslmed.adf1147 37099632

[5] Berthelet J, Wimmer VC, Whitfield HJ, Serrano A, Boudier T, Mangiola S, et al. The site of breast cancer metastases dictates their clonal composition and reversible transcriptomic profile. Sci Adv. 2021;7(28):eabf4408. doi: 10.1126/sciadv.abf4408 34233875 PMC8262813

[6] Di Chiaro P, Nacci L, Arco F, Brandini S, Polletti S, Palamidessi A, et al. Mapping functional to morphological variation reveals the basis of regional extracellular matrix subversion and nerve invasion in pancreatic cancer. Cancer Cell. 2024;42(4):662-681.e10. doi: 10.1016/j.ccell.2024.02.017 38518775

[7] Chung Y, Ha JH, Im KC, Lee JS. Accurate spatial gene expression prediction by integrating multi-resolution features. In: 2024 IEEE/CVF Conference on Computer Vision and Pattern Recognition (CVPR), 2024. 11591–600. doi: 10.1109/cvpr52733.2024.01101

[8] Song AH, Williams M, Williamson DFK, Chow SSL, Jaume G, Gao G, et al. Analysis of 3D pathology samples using weakly supervised AI. Cell. 2024;187(10):2502-2520.e17. doi: 10.1016/j.cell.2024.03.035 38729110 PMC11168832

[9] Almagro-Perez C, Song AH, Weishaupt L, Kim A, Jaume G, Williamson DFK. AI-driven 3D spatial transcriptomics. Arxiv. 2025. doi: 10.48550/arXiv.2502.17761

[10] Hiller JG, Cole SW, Crone EM, Byrne DJ, Shackleford DM, Pang J-MB, et al. Preoperative β-blockade with propranolol reduces biomarkers of metastasis in breast cancer: a phase II randomized trial. Clin Cancer Res. 2020;26(8):1803–11. doi: 10.1158/1078-0432.CCR-19-2641 31754048

